# Expression of Nestin by Neural Cells in the Adult Rat and Human Brain

**DOI:** 10.1371/journal.pone.0018535

**Published:** 2011-04-07

**Authors:** Michael L. Hendrickson, Abigail J. Rao, Omar N. A. Demerdash, Ronald E. Kalil

**Affiliations:** 1 W.M. Keck Laboratory for Biological Imaging, University of Wisconsin–Madison, Madison, Wisconsin, United States of America; 2 Department of Neurological Surgery, Oregon Health and Science University, Portland, Oregon, United States of America; 3 School of Medicine and Public Health, University of Wisconsin–Madison, Madison, Wisconsin, United States of America; 4 Department of Ophthalmology and Visual Sciences, School of Medicine and Public Health, University of Wisconsin–Madison, Madison, Wisconsin, United States of America; University of North Dakota, United States of America

## Abstract

Neurons and glial cells in the developing brain arise from neural progenitor cells (NPCs). Nestin, an intermediate filament protein, is thought to be expressed exclusively by NPCs in the normal brain, and is replaced by the expression of proteins specific for neurons or glia in differentiated cells. Nestin expressing NPCs are found in the adult brain in the subventricular zone (SVZ) of the lateral ventricle and the subgranular zone (SGZ) of the dentate gyrus. While significant attention has been paid to studying NPCs in the SVZ and SGZ in the adult brain, relatively little attention has been paid to determining whether nestin-expressing neural cells (NECs) exist outside of the SVZ and SGZ. We therefore stained sections immunocytochemically from the adult rat and human brain for NECs, observed four distinct classes of these cells, and present here the first comprehensive report on these cells. Class I cells are among the smallest neural cells in the brain and are widely distributed. Class II cells are located in the walls of the aqueduct and third ventricle. Class IV cells are found throughout the forebrain and typically reside immediately adjacent to a neuron. Class III cells are observed only in the basal forebrain and closely related areas such as the hippocampus and corpus striatum. Class III cells resemble neurons structurally and co-express markers associated exclusively with neurons. Cell proliferation experiments demonstrate that Class III cells are not recently born. Instead, these cells appear to be mature neurons in the adult brain that express nestin. Neurons that express nestin are not supposed to exist in the brain at any stage of development. That these unique neurons are found only in brain regions involved in higher order cognitive function suggests that they may be remodeling their cytoskeleton in supporting the neural plasticity required for these functions.

## Introduction

Nestin is a class VI intermediate filament protein expressed in normal and diseased cells in different tissues and organs [1]–[4]. Among neural cells in the developing and adult CNS, nestin expression is thought to occur exclusively in uncommitted neural progenitor cells (NPCs) [5]–[8]. After NPCs differentiate, nestin expression typically is replaced by the expression of neuronal or glial specific markers.

In the normal adult brain, NPCs are found prominently in two “neurogenic” locations, the subventricular zone (SVZ) of the lateral ventricle and the subgranular zone (SGZ) of the dentate gyrus. NPCs in the SVZ and SGZ of the adult brain have been studied intensively [9], [10]. By contrast, comparatively little attention has been paid to the possibility that nestin-expressing neural cells (NECs) may occur outside of the SVZ or SGZ in the adult brain, although recent reports suggest that some microglia may express nestin [11] and that a very small number of GFAP-expressing cells in the neocortex also appear to express nestin [12].

To shed light on this question, we have conducted an extended series of studies in the adult rat and human brain to determine whether NECs occur in regions of the brain other than the SVZ or SGZ. Here we present what we believe to be the first comprehensive report on NECs in the adult rat and human brain. In the rat brain, we have defined four classes of NECs. Class I and Class IV cells are found widely throughout the forebrain, whereas Class II cells are located along the walls of the third ventricle and aqueduct and in the medial wall of the lateral ventricle near its juncture with the third ventricle. Class III cells are found principally in the cholinergic basal forebrain, the corpus striatum and in the CA1-CA3 fields of the hippocampus. In the human brain, we observed Class III cells in the cholinergic basal forebrain and basal ganglia. Supporting some of these results are reports of Class III-like cells in the adult human [13] and rat brain [14], [15].

Double and triple immunostaining revealed that Class III cells express proteins normally associated only with neurons, such as NeuN, βIII-tubulin, ChAT and EAAC1, strongly suggesting that Class III cells are nestin-expressing neurons (NENs). Birth dating studies in the rat involving injections of BrdU for 28 consecutive days showed that NENs were not born in the 28 day period preceding the last BrdU injection. Challenging NENs in the cholinergic basal forebrain with 192-Saporin, a cytotoxic ligand that disrupts ribosomes in cholinergic neurons, indicated that nestin expression does not afford NENs neuroprotection against this type of insult. While further work is required to clarify the role played by nestin expression in NENs, the results presented here demonstrate that nestin expression by a cell of neural lineage in the adult brain is not sufficient by itself to identify the cell as a neural progenitor cell.

## Materials and Methods

### Ethics Statement

#### Research animals

All animal handling and procedures were performed in accordance with protocols for these studies that have been approved by the Institutional Animal Care and Use Committee at the University of Wisconsin-Madison under M00147-0-08-08. All surgery was performed under deep anesthesia and every attempt was made to minimize pain and discomfort.

#### Human brain

Human brain tissue was obtained from the Wisconsin Brain Donor Program which provides brain tissue for scientific research from deidentified donors. The procedures for obtaining the tissue used in these studies and the research that was conducted with the tissue were approved under M-2004-1263 by the Health Sciences Internal Review Board of the University of Wisconsin School of Medicine and Public Health, which determined that the research did not meet the definition of "human subjects" under 45 CFR 46.102(f), and, therefore, did not require consent.

### Research Animals

Thirty-one adult male Sprague-Dawley rats (250–300g) were used in this study. Four of these rats were used as normal controls. The animals were deeply anesthetized with pentobarbital sodium (60 mg/kg) and perfused transcardially with 4% paraformaldehyde in 0.1 M phosphate buffer. The brains of control and experimental rats then were postfixed in 4% paraformaldehyde and subsequently sectioned at a thickness of 40 µm with a vibrating blade microtome (Leica Microsystems).

#### Cell Proliferation

Six rats were used in cell proliferation studies. Three animals received a daily intraperitoneal injection (75 mg/kg) of BrdU (Sigma) for 28 consecutive days, and then were perfused as described above 8 hr after the last BrdU injection. Three additional animals were injected once daily with BrdU (150 mg/kg) for 7 consecutive days and then were perfused 12 hours after the last injection. The brains then were postfixed and sectioned as mentioned.

#### Cortical Lesion

Nine rats received a unilateral lesion of the visual cortex. Following an intramuscular injection of 70 mg/kg of ketamine HCl and 7 mg/kg of xylazine, anesthesia was maintained using 1.0–2.0% isoflurane in oxygen at a flow rate of 300 mL/min. The head was shaved before placement in a stereotaxic holder, and ophthalmic gel was used to protect the corneas from drying. Using aseptic procedures, a midline incision was made to expose the skull. A bone flap was cut to expose the visual cortex and a small aspiration lesion, 1.0–2.0 mm in diameter, 1.0 mm in depth, was made. The scalp wound then was closed and the animals were allowed to recover on a heating pad. Following survival periods of 3, 7 and 14 days (n = 3 at each survival period) the rats were perfused and the brains sectioned as described above.

#### 192-Saporin Injections

Twelve rats were anesthetized and the dorsal surface of the skull was exposed as described above. A burr hole in the skull was made immediately above the intended injection site, and a unilateral injection of 192-Saporin (Advanced Targeting Systems) was made into the lateral ventricle using the following coordinates [16] relative to bregma: anteroposterior, −1.35 mm; mediolateral, 1.80 mm; and dorsoventral, −3.50 mm. Three different concentrations of 192-Saporin, 0.5, 1.0, or 2.0 µg in 6 µL of 0.1 M phosphate buffered saline (PBS), were injected (n = 3 for each concentration) over 10 min. The needle was left in place for 5 min after completion of the injection and then slowly withdrawn. The scalp incision was closed with wound clips, and animals were allowed to recover on a heating pad before being returned to their home cages. Three additional rats received an injection of PBS into the ventricle following the same procedures that were used for the 192-Saporin injections. The animals were allowed to survive for six days and then were perfused and the brains sectioned as described.

### Human Brain

Individuals with a history of psychiatric illness, neurologic disease, or neuropathology were excluded from this study. Following autopsy, brains from four adult males (32–90 years of age) were immersion fixed in formalin. Tissue blocks containing the regions of interest were grossly dissected and subsequently sectioned at a thickness of 40 µm on a vibrating blade microtome (Leica Microsystems).

### Chromogenic Immunohistochemistry

Sections stained for nestin or the low-affinity nerve growth factor receptor, p75(NGFR) were permeabilized by incubating them in 0.2% Triton-X 100 for 15 min and then blocked in 5% normal horse serum for 1 hr. Rat brain sections then were incubated overnight (∼16 hours) at 4°C in a mouse monoclonal antibody raised against nestin at 1∶500 (Rat-401, Millipore) or with a rabbit polyclonal anti-p75(NGFR) antibody at 1∶000 (Millipore). Human brain sections were incubated overnight at 4°C in a mouse monoclonal anti-human nestin antibody at 1∶20 (Abcam). Sections then were incubated for 2 hrs in a biotinylated horse anti-mouse IgG secondary antibody at 1∶500 (Vector Laboratories) or a biotinylated donkey anti-rabbit IgG secondary antibody at 1∶250 (Jackson ImmunoResearch). Next, they were incubated in a horseradish-peroxidase conjugated avidin-biotin complex for 2 hrs (Vectastain Elite ABC Kit, Vector Laboratories). Staining was resolved with untoned, nickel chloride-toned, or cobalt acetate-toned diaminobenzidine (Vector Laboratories), and sections then were dehydrated and coverslipped in Permount (Fisher Scientific). Sections stained for the microglial marker CD11b were not permeabilized. After washing in 0.1 M PBS, the sections were blocked in 4% BSA for 1 hr at RT and then incubated overnight at 4°C in a mouse anti-CD11b antibody (OX42, Millipore) at 1∶250 in 0.1 M PBS with 1% BSA added. Sections then were processed as described above.

### Fluorescence Immunohistochemistry

Sections first were permeabilized by incubation in 0.2% Triton-X 100 for 15 min and then blocked in 5% normal horse serum for 1 hr at RT. Rat brain sections were incubated overnight at 4°C in a rabbit polyclonal anti-nestin antibody at 1∶400 (clone 130; a gift of Ronald McKay, National Institutes of Health). Sections from the human brain were incubated overnight at 4°C with a mouse monoclonal anti-human nestin antibody at 1∶20 (Abcam). We did not notice any difference between the mouse monoclonal and the rabbit polyclonal antibodies in the sensitivity or pattern of nestin staining. A rabbit polyclonal antibody, rather than a mouse monoclonal antibody, was used in staining rat sections for nestin to avoid using two primaries made in the same species when double immunostaining for nestin and cell specific markers (see below).

Sections then were incubated for 2 hrs in a biotinylated donkey anti-rabbit IgG secondary antibody at 1∶250 (Jackson ImmunoResearch) or a biotinylated horse anti-mouse IgG secondary antibody at 1∶500 (Vector). Next, they were incubated for 2 hrs in a horseradish-peroxidase conjugated avidin-biotin complex (Vectastain Elite ABC Kit, Vector). The bound complex was visualized by reaction for 5 min with a tyramide signal amplification substrate conjugated to fluorescein (PerkinElmer Life and Analytical Sciences).

Double immunostaining of rat brain sections for cells co-expressing nestin and one of the following markers: neuronal nuclei (NeuN), βIII-tubulin, glial fibrillary acidic protein (GFAP), vimentin, SOX-2, GST-π, NG2, or doublecortin (DCX) was accomplished by first staining for nestin as described, and then further incubating the sections overnight at 4°C in mouse monoclonal antibodies raised against NeuN (Millipore, 1∶200), βIII-tubulin (Promega, 1∶2000), GFAP (Biogenex, 1∶250) vimentin (Millipore, 1∶200), or SOX-2 (Millipore, 1∶500), or in rabbit polyclonal antibodies raised against NG2 (Millipore, 1∶200), GST-π (MBL International, 1∶10,000) or DCX (Cell Signaling, 1∶500) followed by 2 hrs of incubation in a Cy3-conjugated goat anti-mouse IgG or a goat anti-rabbit IgG secondary antibody at 1∶500 (Jackson ImmunoResearch).

Double immunostaining for nestin and BrdU in rat brain sections was conducted by first staining the sections for nestin using tyramide histochemistry as described above. Next, the sections were treated with 2 N HCl for 1 hr, neutralized for 10 min in 0.1 M sodium borate, and incubated overnight at 4°C in a sheep polyclonal anti-BrdU primary antibody at 1∶200 (Maine Biotechnology Services). The sections then were incubated in a donkey anti-sheep IgG secondary antibody conjugated to Cy3 at 1∶500 (Jackson ImmunoResearch) to resolve BrdU^+^ cells. For NeuN and BrdU staining, NeuN expression was resolved as described above with a Cy2 conjugated secondary antibody at 1∶500, and then the sections were stained for cells that had incorporated BrdU as mentioned.

For nestin and choline acetyltransferase (ChAT) double-staining in rat brain, sections first were permeabilized by incubation in 0.2% Triton-X 100 for 15 min and then blocked in 5% normal donkey serum for 1 hr. They then were incubated overnight at 4°C in mouse monoclonal anti-nestin primary antibody at 1∶200 (Rat-401, Millipore), followed by 2 hrs in a biotinylated horse anti-mouse IgG secondary antibody at 1∶250 (Vector Laboratories). Sections next were incubated for 2 hrs in Cy2-conjugated streptavidin at 1∶250 (Jackson ImmunoResearch). The sections were further incubated overnight at 4°C in a goat polyclonal anti-ChAT primary antibody at 1∶200 (Millipore), followed by 2 hrs in a Cy3-conjugated donkey anti-goat IgG secondary antibody at 1∶500 (Jackson ImmunoResearch).

Triple immunostaining in rat brain sections for nestin, NeuN, and the neuronal glutamate transporter EAAC1 or for nestin, NeuN and ChAT was conducted by staining sections first for nestin with a rabbit polyclonal primary antibody and tyramide histochemistry as mentioned, then for NeuN as described, except that antibody binding was resolved with a Cy5-conjugated donkey anti-mouse IgG secondary antibody at 1∶500 (Jackson ImmunoResearch). Sections then were incubated overnight at 4°C in a goat polyclonal anti-EAAC1 primary antibody at 1∶4000 (Millipore) or a goat polyclonal anti-ChAT primary antibody at 1∶200 (Millipore), followed by 2 hrs in a Cy3-conjugated donkey anti-goat IgG secondary antibody at 1∶250 (Jackson ImmunoResearch).

For nestin and ChAT double-staining in human brain, sections were stained for nestin as above, and further incubated overnight at 4°C in the goat polyclonal anti-ChAT primary antibody at 1∶250 (Millipore) followed by 2 hrs in a Cy3-conjugated donkey anti-goat IgG secondary antibody at 1∶250 (Jackson ImmunoResearch). As a last step, the marked autofluorescence that is evident in cells of the aged human brain was suppressed by treating the sections for five minutes with an autofluorescence reduction agent containing Sudan Black (Millipore) (Figure S1).

Rat and human brain sections were mounted from tap water, and coverslipped in glycerol. All of the secondary antibodies used for chromogenic or fluorescence labeling were highly cross-adsorbed and did not recognize endogenous rat IgG or human IgG present in the tissue sections, nor did they cross-react with any of the IgGs contributed by the primary or secondary antibodies that were used.

### Brightfield and Confocal Microscopy

Brightfield microscopy was performed with a Nikon upright microscope, and images were collected with a Nikon digital camera. Confocal microscopy was performed with a Bio-Rad Radiance 2100 MP Rainbow laser scanning confocal system coupled to a Nikon TE2000 inverted microscope. All images were collected sequentially with the appropriate emission filters using excitation laser lines of 488 nm for fluorescein and Cy2, 543 nm for Cy3, and 637 nm for Cy5. Each image was collected as a single optical section using a 60X objective (NA 1.40), giving a calculated optical slice thickness of ∼2.0 µm. To further verify double- and triple-labeling observed in the XY plane, cells were imaged through the YZ plane at 0.50-µm intervals. Images were pseudocolored green for fluorescein and Cy2, red for Cy3, and blue for Cy5. The contrast/brightness adjustment and layout of all figures were performed using Adobe Photoshop CS3 (Adobe Systems).

### Quantitative Analysis

#### Cell Soma Size of Class I and Class II Cells and CD11b^+^ Cells

The outlines of at least 50 cell somas in nestin stained sections (Class I and Class II cells) and in sections stained for CD11b, a cell surface antigen that marks microglial cells, were traced at 1000X with the aid of a drawing tube. The drawings then were digitized and the area within each tracing was computed with an image analysis program (Image J, NIH). The mean cross-sectional, cell soma area of Class I cells was compared with the mean cell soma area of CD11b^+^.

#### Class I Cell and CD11b^+^ Cell Density in the Rat Neocortex

Class I cells and CD11b^+^ cells were counted with the aid of an eyepiece grid that subtended 4.2×10^4^ µm^2^ at 400X. The grid was first positioned at the dorsal surface of the cortex and then moved ventrally in steps until the depth of the cortex had been traversed. The grid then was positioned laterally one step and moved dorsally in stepwise fashion. This procedure was continued until 30 grid fields, 1.3×10^6^ µm^2^, had been surveyed and all Class I cells or CD11b^+^ cells had been counted.

#### NEN Cell Soma Size and Percentages

The cross-sectional areas of cell somas of 50 NENs with a visible nucleus and of 50 nearby conventional neurons were drawn from single, cresyl-violet stained sections through the medial septal nucleus (MSN), the nucleus of the diagonal band (NDB) and the corpus striatum (CS) in each of three brains. Cell soma outlines were traced in the plane of the nucleus at 1000X with the aid of a drawing tube. Samples were collected with an eyepiece grid that was moved in stepwise fashion until the requisite number of cell somas had been traced. The mean cross-sectional, cell soma area of NENs was computed as described above and compared with the mean cross sectional cell soma area of neighboring neurons.

The percentage of NENs in the MSN, NDB, CS, and the CA1, CA2, and CA3 fields of the hippocampus was determined in three adult rats by counting NeuN^+^/nestin^+^ and NeuN^+^/nestin^-^ cells in confocal images (400X) of sections stained as above with fluorophore conjugated antibodies. Three to five representative fields, each 5.8×10^4^ µm^2^, in each region in each of three animals were examined, and all of the NeuN^+^/nestin^-^ and NeuN^+^/nestin^+^ cells were counted until at least 100 cells had been counted in each region in each animal. The counts were made by two independent observers and then were averaged. Only cells that displayed a clear nucleus, and in most instances a nucleolus, were counted. The percentage of NENs in each brain region in each brain then was determined by dividing the total number of NeuN^+^/nestin^+^ cells by the total number of NeuN^+^ cells.

#### Cell Survival Following Injections of 192-Saporin

We determined the number of ChAT^+^/nestin^-^ and of ChAT^+^/nestin^+^ neurons in the MSN and NDB in normal control rats and in rats that were injected intraventricularly with 0.5, 1.0 or 2.0 µg 192-Saporin in 6 µL of PBS (n = 3 for each group). Three coronal sections (40 µm) from each brain, located 0.5, 1.0, and 1.5 mm rostral to the interhemispheric limb of the anterior commissure, containing both the MSN and NDB were stained for nestin and ChAT as described above. As the border between the MSN and the NDB is not easily distinguished cytologically, we considered cells located dorsal to the anterior commissure to belong to the MSN and cells located ventral to the commissure to belong to the NDB. Images at 300X of twelve non-overlapping fields from the MSN and from the NDB in each section were collected using a confocal microscope. At least 100 cells per region per animal were counted by two observers and the counts then were averaged. To exclude stained cell fragments from the counts, only cells stained for ChAT in which a nucleus was clearly evident were counted. Next, the number of ChAT^+^ cells in each image that also expressed nestin was determined. A neuron was considered to be ChAT^+^/nestin^+^ if staining for nestin was clearly above background in at least half the area of the cell soma as delineated by the ChAT staining. The mean percentage of cells co-expressing ChAT and nestin in each of the 192-Saporin-treated groups of rats was compared with the corresponding percentage of cells in the control group of rats.

### Statistical Analysis

All mean values are expressed ± the standard error of the mean. Comparisons between means were evaluated with unpaired Student' two tailed *t*-tests. Differences between means were considered significant when they exceeded p<0.01. In comparing mean cell soma sizes of NENs with that of conventional neurons in the MSN, NDB and CS, differences were evaluated statistically using the mean cell soma area of each group of cells for each animal as a single value.

## Results

### Nestin Expression in the Adult Rat Brain

As has been reported by many others, nestin expression was observed in regions of the adult brain considered to be neurogenic, e.g., the SVZ and the SGZ [17], [18]. In the SVZ, cells expressing nestin lined the lateral walls of the anterior lateral ventricles, and nestin^+^ cells were observed as they left the SVZ in the rostral migratory stream to course mediolaterally over the dorsal surface of the corpus striatum, immediately subjacent to the corpus callosum, en route to the olfactory bulb (Figure S2). In the dentate gyrus, nestin^+^ processes were found scattered throughout the SGZ. However, nestin immunostaining also revealed four distinct classes of nestin-expressing neural cells in regions of the brain not thought to be neurogenic.

#### Class I cells

**Figure 1:**
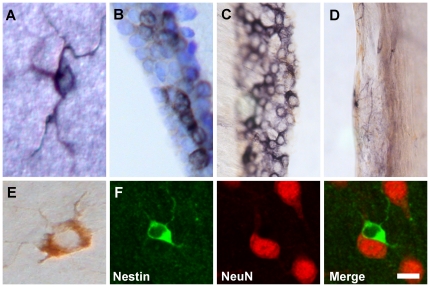
Class I, II and IV cells. A typical Class I cell, immunostained chromogenically for nestin, with a very small cell soma and few sparse processes (**A**). Class II cells, stained for nestin chromogenically and counter-stained with cresyl violet, clustered in the wall of the third ventricle (**B**). Class II cells stained for nestin chromogenically in the medial wall of the lateral ventricle near its merger with the third ventricle (**C**). The lateral wall of the lateral ventricle directly opposite the medial wall shown in **C.** Note the absence of Class II cells in the lateral wall (**D**). Class IV cell stained chromogenically. Note the small number of very fine processes (**E**). Immunofluorescence staining of a Class IV cell (green) and three NeuN^+^ neurons (red) (**F**). The merged image in the right panel shows that the Class IV cell abuts one of the neurons. Scale bar: 5 µm (**A**) and (**E**), 10 µm (**B**), 20 µm (**C**) and (**D**), 15 µm (**F**).

Class I cells are scattered at low density throughout the adult rat forebrain. These cells have very small cell somas, approximately 23 µm^2^, that support three to four processes that frequently branch close to the cell soma (Figure 1A). In sections that had been stained for vimentin, NG2 or DCX or any of the markers associated with neurons and glial cells such as NeuN, βIII-tubulin (neurons), GFAP (astroglia), or GST-π (oligodendroglia) we never observed any cells that resembled Class I cells.

**Figure 2:**
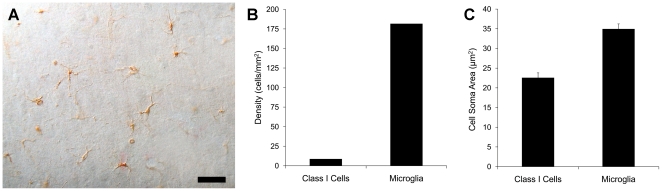
Cell soma size and density of Class I cells and CD11b^+^ microglia. The number of Class I nestin^+^ cells is increased twentyfold in the immediate vicinity of a cortical lesion (**A**). Histograms showing the density of nestin^+^ Class I cells and CD11b^+^ microglia in the neocortex of a normal rat (**B**). The density of microglial cells is more than fifteen times greater than that of Class I cells. Histograms showing the mean cell soma cross sectional area for nestin^+^ Class I cells and CD11b^+^ microglia (**C**). The mean cell soma size of microglial cells is 1.5 times that of Class I cells. The difference in mean cell soma size between Class I cells and microglia is significant (p <.001, two tailed t-test). Scale bar: 20 µm.

Following a cortical injury, Class I cells proliferate more than 20 fold in the immediate vicinity of the injury (Figure 2A). This proliferation in response to an injury suggested that Class I cells might be related to microglia. However, sections stained for the microglial marker CD11b demonstrated that CD11b^+^ microglia are 15 times as dense in the neocortex as Class I cells (Figure 2B), and that the mean cell soma area of microglia is 35 µm^2^, 1.5 times greater than the mean cell soma area of Class I cells (Figure 2C). These results indicate that Class I cells are not equivalent to the microglia that are resolved by CD11b staining.

#### Class II cells

In contrast to the widespread distribution of Class I cells, Class II cells are found chiefly in the walls of the third ventricle (Figure 1B) and aqueduct, and in the medial wall of the lateral ventricle immediately rostral to its merger with the third ventricle (Figure 1C). Class II cells have small round cell somas, approximately 65 µm^2^. Unlike nestin^+^, third ventricle tanycytes [19]-[22], Class II cells appear to support few or no processes (Figure 1B). In three rats that received a daily intraperitoneal injection of BrdU (150 mg/kg) for 7 consecutive days, we did not detect any BrdU-labeled Class II cells.

#### Class IV cells

**Figure 3:**
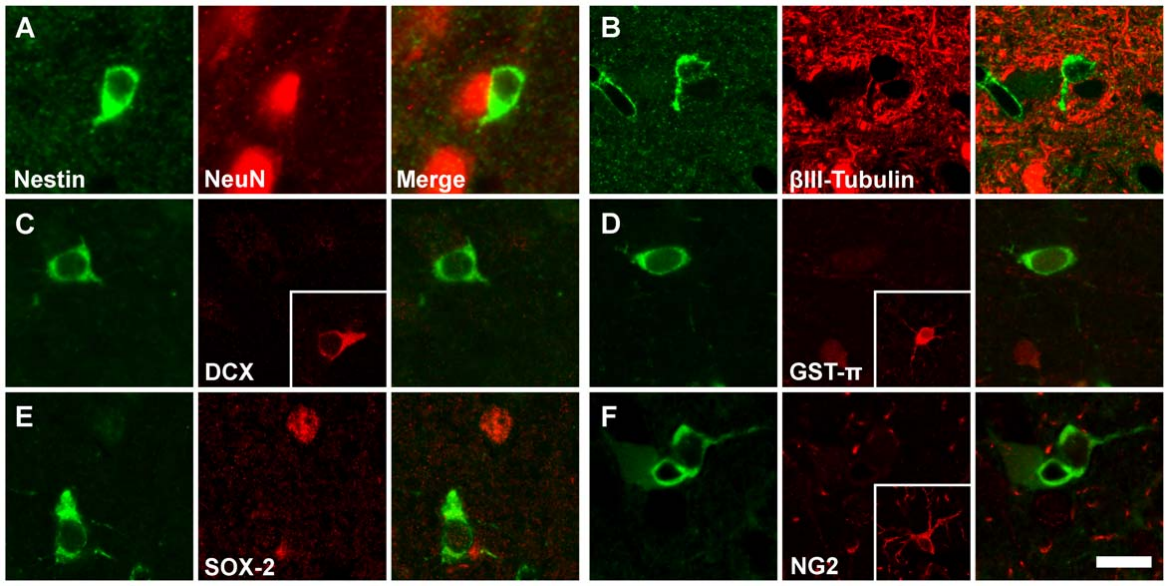
Class IV cells do not express typical neural cell markers. Class IV cells, which express nestin (green), do not express the neural cell markers (red): NeuN (**A**), βIII-tubulin (**B**), doublecortin (**C**), GST-π (**D**), SOX-2 (**E**), or NG2 (**F**). Insets show images of cells expressing the specific marker collected using the same confocal acquisition settings. Scale bar: 15 µm (30 µm for the insets).

Class IV cells have cell somas that are approximately 115 µm^2^ on average, with a large prominent nucleus, scant cytoplasm and three to eight fine processes that rarely extend more than 10 µm from the cell soma (Figure 1E). Like Class I cells, Class IV cells are widely distributed throughout the brain. However, the micro-distribution of Class IV cells is unique in that nearly 80% of Class IV cells are located in a satellite position immediately adjacent to a neuron as confirmed by NeuN expression (Figure 1F). This location might suggest that Class IV cells are oligodendrocytes that express nestin, but this possibility is unlikely because double staining for nestin and GST-π, an oligodendrocyte marker, indicated that Class IV cells do not express GST-π (Figure 3). Nor are Class IV cells likely to be oligodendrocyte precursors as they are not NG2^+^. Moreover, Class IV cells do not express GFAP or SOX2, suggesting that they are not astrocytes or neural progenitor cells, or DCX, implying that they are not newly generated neurons, or NeuN or βIII-tubulin, indicating that they are not mature neurons (Figure 3).

#### Class III cells

**Figure 4:**
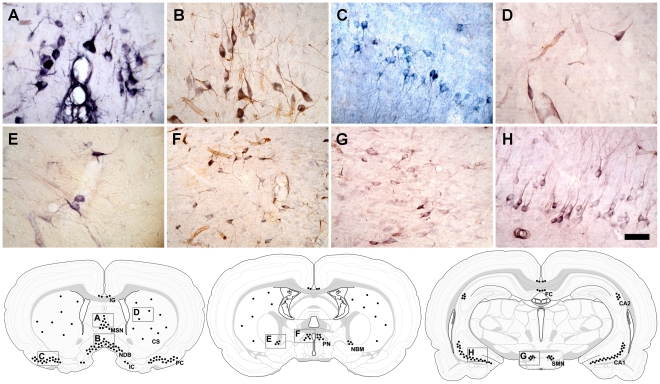
Class III cells. Class III cells immunostained chromogenically for nestin are found densely distributed in the nucleus of the diagonal band (**A**) and medial septal nucleus (**B**). In the piriform cortex, Class III cells are lightly stained and found predominantly in layer II (**C**). Class III cells are scattered throughout the corpus striatum (**D**) and basal nucleus of Meynert (**E**), and are prominently displayed in the median preoptic nucleus (**F**) and the submamillothalamic nucleus of the hypothalamus (**G**). Class III cells found in the CA1, CA2, and CA3 fields of the hippocampus reveal a stereotyped structure consistent with that of pyramidal cells (**H**). Scale bar: 50 µm. Coronal drawings at three AP levels of the adult rat brain summarizing the regions in which NENs (black dots) are found and their relative distribution. The letters in each drawing correspond to the lettered panels **A**–**H**. NENs also are found in the islands of Calleja (**IC**), induseum griseum (**IG**), fasciola cinerea (**FC**) and preoptic nuclei (**PN**). Abbreviations: Corpus striatum (**CS**)**,** hippocampal fields (**CA1, CA2**), medial septal nucleus (**MSN**), basal nucleus of Meynert (**NBM**)**,** nucleus of the diagonal band (**NDB**), submamillothalamic nucleus (**SMN**).

Class III cells have medium to large cell somas, approximately 180–230 µm^2^ depending upon their location, that support nestin^+^ processes of varying complexity that also stained for nestin. In general, the cytoarchitecture of Class III cells revealed by nestin staining closely resembled that of the predominant neurons in the anatomical regions where Class III cells were found. Class III cells are not found in the neocortex, but were observed in the following regions of the adult brain, none of which are thought to be neurogenic: the corpus striatum, nucleus of the diagonal band (NDB), medial septal nucleus (MSN), piriform cortex, basal nucleus of Meynert, the pyramidal cell layer of the hippocampus, and in the following areas of the hypothalamus; the lateral preoptic nucleus, medial preoptic area, anterodorsal preoptic nucleus, the median preoptic nucleus, and the submammilothalamic nucleus. Class III cells also were found in the medial habenular nucleus, islands of Calleja, fasciola cinerea, and induseum griseum (Figure 4).

Class III cells in the NDB (Figure 4A) and MSN (Figure 4B) had medium to large cell somas, 231±9 µm^2^ (n = 3) and 182 ±10 µm^2^ (n = 3), respectively, that stained more darkly than Class III cells in other regions of the brain. The processes of these cells also often were stained darkly, sometimes delineating secondary and tertiary branches. In contrast, Class III cells found in layer II of the piriform cortex stained lightly for nestin with limited staining of proximal processes (Figure 4C). Class III cells were scattered throughout the corpus striatum (Figure 4D), had medium to large cell somas, 203±2 µm^2^, similar in size to those of Class III cells in the MSN and NDB, and were the largest cells seen in the corpus striatum. These cells showed nestin immunoreactivity primarily in their cell somas and in proximal processes, with only occasional staining evident in secondary and tertiary processes. Class III cells in the basal nucleus of Meynert (Figure 4E) had medium-sized somas, and showed light staining, clearly distinguishing them from cells in the adjacent corpus striatum. In the median preoptic nucleus (Figure 4F), and the in the submammilothalamic nucleus (Figure 4G) of the hypothalamus, Class III cells typically had medium-sized somas and showed nestin staining predominantly in cell somas and in primary processes.

Class III cells in the pyramidal cell layer of the hippocampus were not distributed uniformly rostro-caudally, but instead were found in clusters. The somas of hippocampal Class III cells displayed strong nestin staining, and in some cells long processes that appeared to be dendrites extending into deeper layers of the hippocampus (Figure 4H) also were nestin^+^. In all of the CA fields of the hippocampus, CA1-CA3, Class III cells were structurally similar to pyramidal cells. The structural similarities between Class III cells and neurons led us to investigate whether Class III cells were in fact neurons as determined by expression of recognized neuronal markers.

### Class III Cells Express Neuronal Markers and Neurotransmitter-Associated Proteins

**Figure 5:**
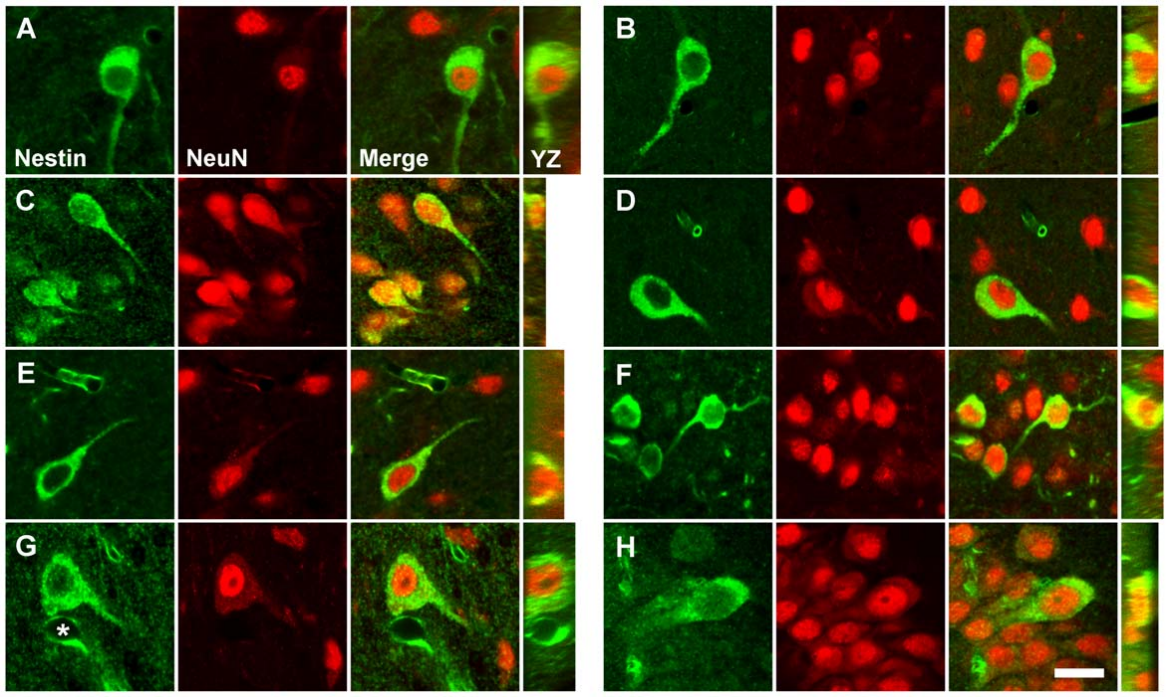
Class III cells are NeuN^+^ nestin expressing neurons (NENs). Confocal images of cells double stained for nestin (green) and neuronal nuclei (NeuN, red) reveal that all nestin^+^ cells in the regions described in Figure 4 also express NeuN, a marker associated exclusively with neurons. The cytological distribution of NeuN expression was identical in both NeuN^+^/nestin^+^ cells (NENs) and NeuN^+^/nestin^-^ cells. Many NeuN^+^/nestin^-^ nuclei are seen in close proximity to NeuN^+^/nestin^+^ cells. Shown are representative NeuN^+^/nestin^+^ NENs from the nucleus of the diagonal band (**A**), the medial septal nucleus (**B**), piriform cortex (**C**), corpus striatum (**D**), basal nucleus of Meynert (**E**), median preoptic nucleus (**F**), submamillothalamic nucleus (**G**), and CA2 field of the hippocampus (**H**). The asterisk in Figure 5G denotes the lumen of a blood vessel, cut in cross-section, surrounded by nestin^+^ endothelial cells, a configuration that could be mistaken for a nestin^+^ cell. Vertical images taken through the YZ plane of the tissue section confirm the co-localization of nestin and NeuN evident in the XY plane. Scale bar: 20 µm.

**Figure 6:**
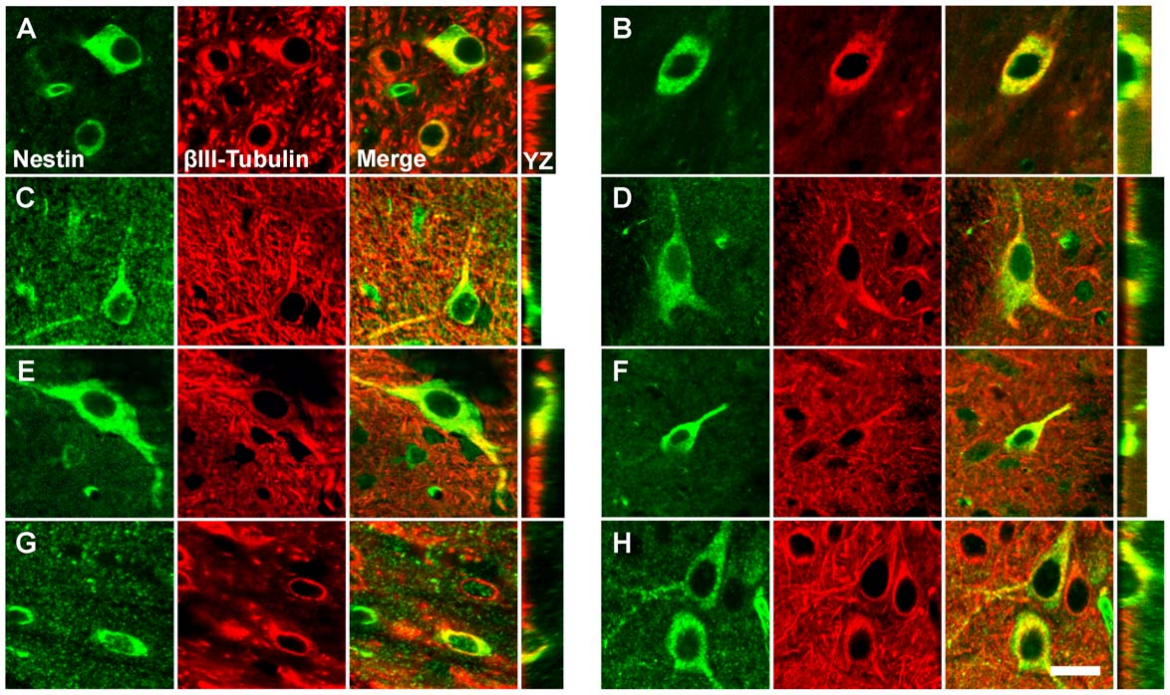
NENs express βIII-tubulin. Confocal images of cells double-stained for nestin (green) and βIII-tubulin (red) demonstrate that all nestin^+^ cells in the regions described in Figures 4 and 5 also express βIII-tubulin, a cytoskeletal marker associated exclusively with neurons. The cytological distribution of βIII-tubulin expression was identical in both βIII-tubulin^+^/nestin^+^ cells (NENs) and βIII-tubulin^+^/nestin^-^ cells. Shown are representative βIII-tubulin^+^/nestin^+^ NENs from the nucleus of the diagonal band (**A**), the medial septal nucleus (**B**), piriform cortex (**C**), corpus striatum (**D**), basal nucleus of Meynert (**E**), median preoptic nucleus (**F**), submamillothalamic nucleus (**G**), and CA2 field of the hippocampus (**H**). Vertical images taken through the YZ plane of the tissue section confirm the co-localization of nestin and βIII-tubulin evident in the XY plane. Scale bar: 20 µm.

Double-immunofluorescencent staining revealed that all Class III cells in the regions described above also express the neuronal markers NeuN (Figure 5) and βIII-tubulin (Figure 6), but Class III cells do not express vimentin or GFAP (data not shown). NeuN immunoreactivity in both nestin^+^ and nestin^-^ neurons was strong in the nucleus, absent in the nucleolus, and weak yet consistently present in the cytoplasm of the cell soma. Sometimes the NeuN staining extended into the proximal portions of processes, consistent with the distribution of NeuN described by Mullen et al. [23]. βIII-tubulin expression was strongest in the periphery of the cell soma and in the proximal portion of processes. The morphological characteristics of cells co-expressing nestin and a neuronal marker and the cytological distribution of the markers within these cells did not differ from that of surrounding neurons that did not express nestin.

**Figure 7:**
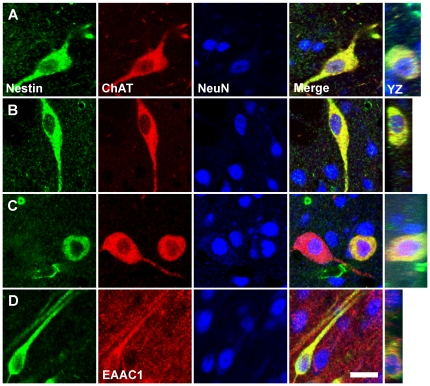
NENs express neurotransmitter-associated proteins. Triple immunofluorescent staining of cells in the corpus striatum and in regions of the cholinergic basal forebrain confirm that all nestin^+^ cells (green) in these areas also express both NeuN (blue) and choline acetyltransferase (ChAT, red), a marker of cholinergic neurons. Representative cholinergic NENs in the medial septal nucleus (**A**), corpus striatum (**B**), and basal nucleus of Meynert (**C**) are shown. All ChAT^+^ cells, whether nestin^+^ or nestin^-^, express NeuN. All nestin^+^ cells in the pyramidal cell layer of the hippocampus express both NeuN and the neuronal glutamate transporter EAAC1 (red), a marker of glutamatergic neurons. EAAC1^+^ NENs in all three hippocampal fields are structurally similar to the NEN shown in CA1 (**D**). Vertical images taken through the YZ plane of the tissue section confirm the co-localization of nestin and ChAT or nestin and EAAC1 evident in the XY plane of a given NEN. Scale bar: 20 µm.

Double and triple immunocytochemical staining of more than 1000 Class III cells demonstrated that all of the Class III cells in the NDB, MSN (Figure 7A), basal nucleus of Meynert (Figure 7B), and corpus striatum (Figure 7C) expressed choline acetyltransferase (ChAT). However, approximately 70% of the ChAT^+^ neurons in the MSN and NDB did not express nestin. In reaching this conclusion, care was taken to exclude counting blood vessels cut in cross-section, because blood vessels, with their nestin^+^ endothelial cell walls and apposed nestin^+^ pericytes [1], [24], sometimes resemble nestin^+^ neural cells when cut transversely in sections stained for nestin (asterisk, Figure 5G). In both Class III cells and ChAT^+^/nestin^-^ cells, the cytological distribution of ChAT immunoreactivity was similar; prominent in the cytoplasm of cell somas, and often extending into the proximal processes.

All Class III cells in the CA fields of the hippocampus expressed the neuronal glutamate transporter EAAC1 (Figure 7D), identifying these cells as glutamatergic. EAAC1 immunoreactivity was present in the cytoplasm of cell somas, extended into the proximal portions of processes, and appeared punctate and vesicular. The neuron-like cytoarchitecture of Class III cells in all of the regions in which they have been observed, and the expression by these cells of NeuN and βIII-tubulin, and ChAT or EAAC1 strongly suggests that Class III cells are neurons. Therefore, we have named Class III cells “nestin expressing neurons” or “NENs,” and refer to them by this name or acronym in the text that follows.

### Cell Soma Size of NENs

**Figure 8:**
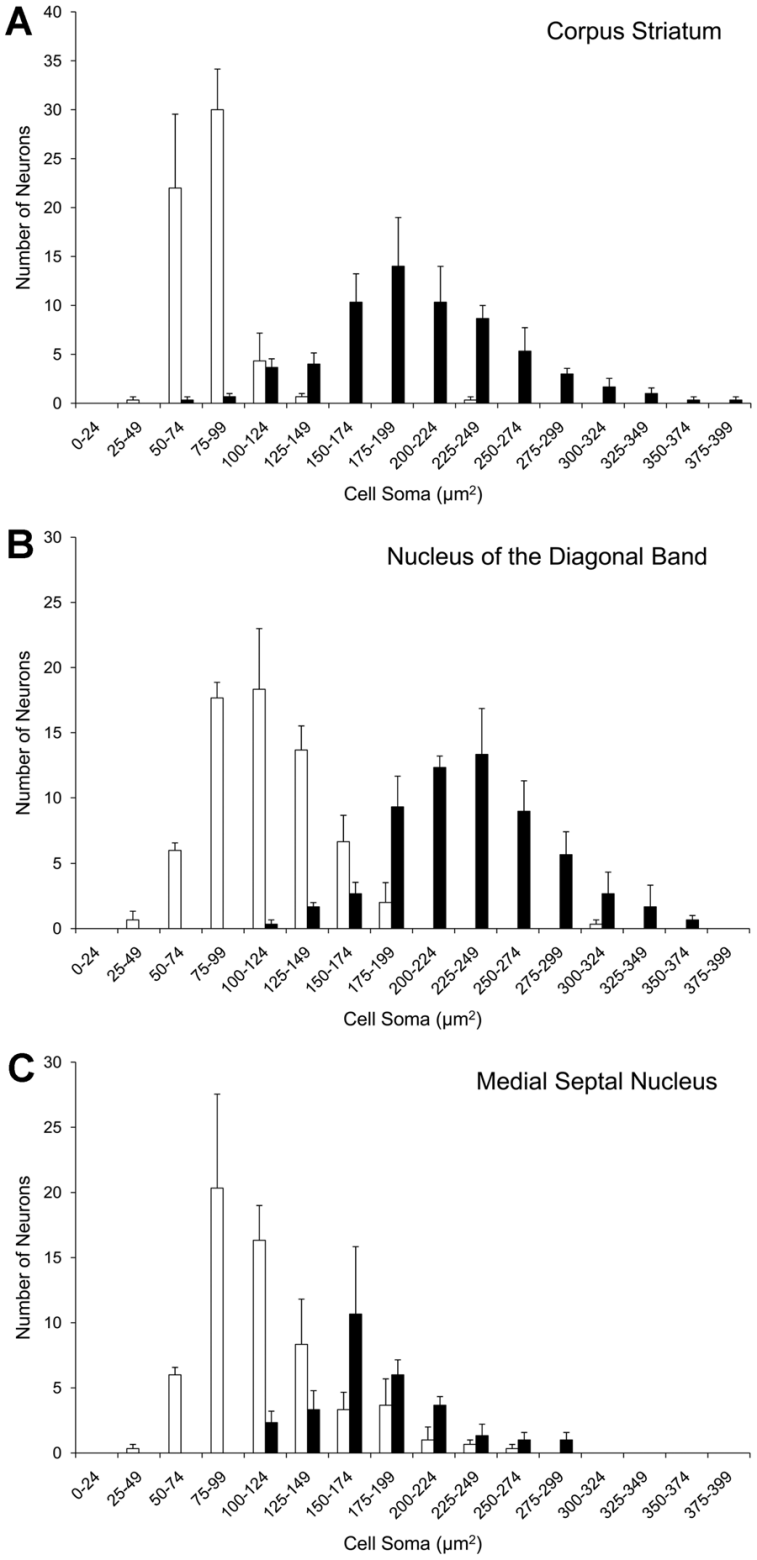
Cell soma size of NENs. Histograms showing the distribution of cell soma cross-sectional areas of NENs (black bars) and non-NENs (white bars) in the corpus striatum (**A**), the nucleus of the diagonal band (**B**) and the medial septal nucleus (**C**). In each of these regions, the mean cell soma size of NENs is significantly greater than the mean cell soma size of non-NENs (see Table 1), and NENs also are the largest cells in each region.

The mean cell soma sizes of NENs in the corpus striatum, NDB and MSN were significantly greater (p<.01, two-tailed *t*-test) than those of conventional neurons in the same regions (Table 1, Figure 8). The difference in cell soma size was particularly pronounced in the corpus striatum where the mean cross-sectional area of NEN cell somas was approximately 2.5 times greater than that of typical neurons in the corpus striatum.

**Table 1:** Cell soma size (µm^2^) of NENs (nestin^+^/NeuN^+^) and non-NENs (nestin^-^/NeuN^+^).

Animal	CS		NDB		MSN	
	NEN	Non-NEN	NEN	Non-NEN	NEN	Non-NEN
1	206	77	220	116	184	114
2	201	75	224	113	164	98
3	201	89	249	115	198	129
Mean±SE	203±2	80±5	231±9	114±1	182±10	114±9

Corpus striatum (**CS**); nucleus of the diagonal band (**NDB**); medial septal nucleus (**MSN**).

### Proportions of NeuN^+^ Cells That Also Are NENs

Since all NENs express NeuN, we calculated the percentage of NENs (NeuN^+^/nestin^+^ cells) in six regions of the brain by counting the number NENs in a defined area and dividing the total number of cells counted by the total number of neurons (NeuN^+^ cells) in the same area (Table 2). The following percentages (mean ± s.e., n = 3) of NENs were determined: 2.0±0.3 in the corpus striatum, 12.0±1.1 in the MSN, 15.3±1.4 in the NDB, and 11.4±0.7, 15.0±4.3, and 29.5±3.6 in those regions of CA1, CA2, and CA3 fields of the hippocampus, respectively, in which NENs were observed.

**Table 2:** NENs (nestin^+^/NeuN^+^) as a percentage of all neurons (NeuN^+^).

Animal	CS	NDB	MSN	CA1	CA2	CA3
1	2.7%	14.4%	14.0%	12.0%	7.9%	29.3%
2	1.6%	13.5%	10.2%	12.2%	14.2%	23.4%
3	1.7%	18.1%	11.7%	10.0%	22.9%	35.9%
Mean±SE	2.0±0.3%	15.3±1.4%	12.0±1.1%	11.4±0.7%	15.0±4.3%	29.5±3.6%

Corpus striatum (**CS**); nucleus of the diagonal band (**NDB**); medial septal nucleus (**MSN**); hippocampal fields (**CA1, CA2, CA3**).

### Are NENs Recently Born?

**Figure 9:**
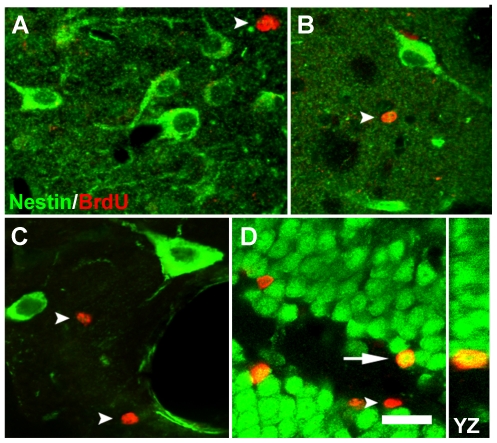
NENs are not recently born. In adult rats given a single injection of BrdU (75 mg/kg) daily for 28 days and sacrificed 8 hours after the last injection, no NENs were labeled with BrdU, such as those seen in the CA1 field of the hippocampus (**A**), corpus striatum (**B**) and nucleus of the diagonal band (**C**). By contrast, BrdU^+^ cell nuclei were observed in each of these regions (arrowheads, **A**–**C**). In the granule cell layer of the dentate gyrus, some NeuN^+^ neurons displayed BrdU^+^ nuclei (arrow, **D**), whereas other BrdU^+^ nuclei were observed in cells that were NeuN^−^ (arrowhead, **D**). Vertical image taken through the YZ plane of the tissue section confirms the NeuN and BrdU co-localization evident in the XY plane. Scale bar: 20 µm (**A**–**C**), 30 µm (**D**).

Given the well-established link between nestin, neural progenitor cells and neurogenesis, we asked whether NENs are recently born, i.e., did they divide within a prior four week period? In the brains of adult rats that received daily intraperitoneal injections of BrdU (75 mg/kg) for 28 days prior to sacrifice, no BrdU^+^ NENs were observed (Figures 9A–C). By contrast, strong BrdU immunoreactivity was seen among dense nestin immunoreactivity in the SVZ and rostral migratory stream (Figure S2), and in the SGZ of the dentate gyrus. In the SVZ and rostral migratory stream (RMS) BrdU^+^ nuclei frequently were observed in nestin^+^ cells, while in the SGZ, BrdU^+^ nuclei often were seen within NeuN^+^ cells (Figure 9D). These results demonstrate that our failure to identify any BrdU^+^ NENs was not the result of inadequate staining for BrdU, but instead indicates that NENs were not generated during the 28 day period when BrdU was administered daily.

### Is the Expression of Nestin by NENs Neuroprotective?

To investigate the possibility that the expression of nestin by NENs might provide a neuroprotective effect, we administered the immunotoxin 192-Saporin. The toxin consists of a ribosome-inactivating protein coupled to a monoclonal antibody directed against the p75(NGFR) nerve growth factor receptor. As a result, 192-Saporin selectively destroys cells expressing the p75(NGFR), which includes most of the cholinergic neurons in the basal forebrain. After confirming immunocytochemically that NENs, which were identified by the co-expression of nestin and ChAT, express p75(NGFR), we injected 192-Saporin unilaterally into the lateral ventricle of adult rats at three concentrations, 0.5, 1.0, and 2.0 µg in 6.0 µL of PBS. The treated rats were allowed to survive for 6 days before double-staining the brains for nestin and ChAT. We then determined the number of NENs in the MSN and NDB as a percentage of all ChAT^+^ neurons in these nuclei in treated rats and in rats that had received a control injection of PBS.

We hypothesized that if the expression of nestin in ChAT^+^ cells protects the cells from the toxic effects of 192-Saporin, then we should observe a higher percentage of ChAT^+^/nestin^+^ cells in 192-Saporin-treated brains than in controls that received injections of PBS. Specifically, If NENs in the MSN and NDB are protected against the toxic effects of 192-Saporin, relative to nestin^-^/ChAT^+^ cells, then it follows that the percentage of NENs with respect to the surviving ChAT^+^ neurons in the MSN and NDB in treated animals will be greater than the comparable percentage of NENs in control animals.

**Figure 10:**
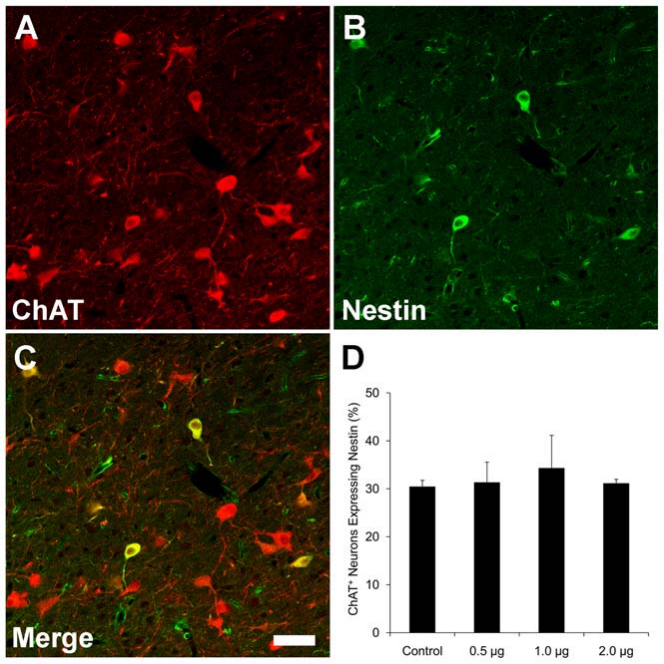
Nestin expression by NENs does not protect against 192-Saporin cytotoxcity. Cells in the nucleus of the diagonal band stained for choline acetyltransferase (ChAT) (**A**) and nestin (**B**). Cells expressing both ChAT and nestin appear yellow in the merged image (**C**)**.** The number of nestin^+^/ChAT^+^ neurons is expressed as a percentage of the ChAT^+^ neurons in the nucleus of the diagonal band and medial septal nucleus in control rats, n = 3, and in rats that received an injection of the toxic ligand 192-Saporin at one of three doses, 0.5 µg, 1.0 µg, or 2 µg in 6 µL of PBS, n = 3, each dose, (**D**). Note that the percentage of nestin^+^/ChAT^+^ neurons remains essentially unchanged at each dosage of 192-Saporin, indicating that nestin expression does not afford any neuroprotection against the cytotoxcity induced by 192-Saporin. Scale bar: 50 µm.

However, there was no significant difference in the percentage of ChAT^+^/nestin^+^ neurons in the MSN and NDB in rats treated with any of the doses of 192-Saporin that were administered in comparison with the percentage of ChAT^+^/nestin^+^ cells observed in the MSN and NDB in rats that had received control injections of PBS (Figures 10 and S3). We therefore conclude that the expression of nestin in a subset of ChAT^+^ neurons in the MSN and NDB does not afford these cells protection from the effects of the cytotoxin 192-Saporin.

### Nestin Expressing Neurons in the Adult Human Basal Forebrain and Related Regions

**Figure 11:**
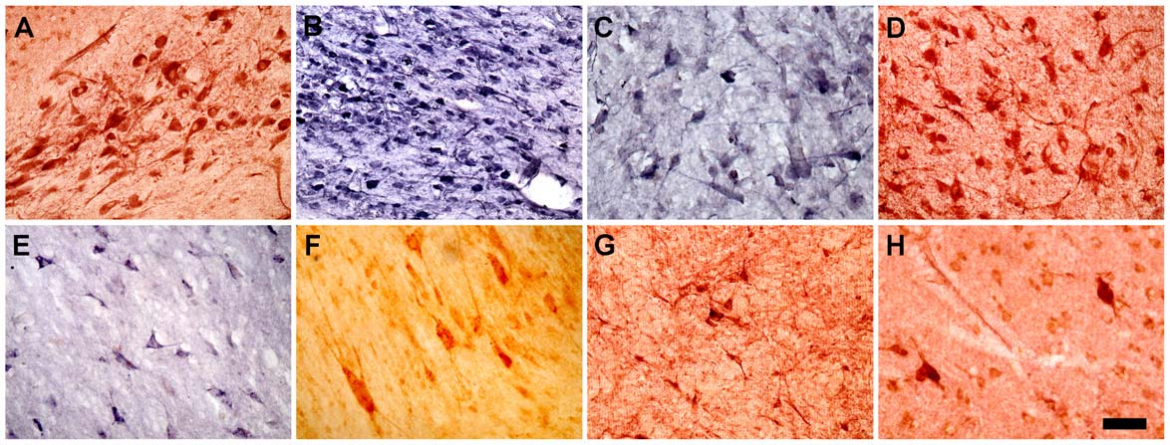
NENs in the human forebrain. NENs are present in a number of regions in the human forebrain, including the basal nucleus of Meynert (**A**), supraoptic nucleus (**B**), paraventricular hypothalamic nucleus (**C**), nucleus of the diagonal band (**D**), septal nucleus (**E**), lateral division of the bed nucleus of the stria terminalis (**F**), globus pallidus (**G**), and putamen (**H**). Scale bar: 100 µm (**A, B, D, G**) and 50 µm (**C, E, F, H**).

To shed light on the distribution of Class III-like cells in the adult human brain, postmortem tissue samples from the basal forebrain and basal ganglia of four adult male subjects (32–90 years of age) were studied. We observed a class of nestin-expressing cells with medium to large cell somas that displayed cytoarchitectural features characteristic of mature neurons, and were comparable structurally to the Class III cells described in the rat brain that we have termed nestin-expressing neurons, NENs. Class III-like cells were seen in the human basal forebrain and in related areas that are not thought to be neurogenic, such as the basal nucleus of Meynert, supraoptic nucleus, paraventricular nucleus of the hypothalamus, NDB, septal nucleus, lateral division of the bed nucleus of the stria terminalis, globus pallidus, and putamen (Figures 11 and S4).

**Figure 12:**
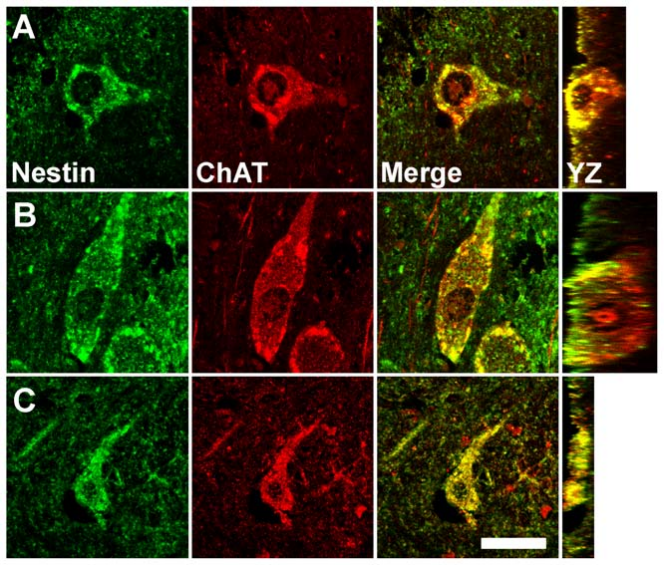
NENs in the human brain express choline acetyltransferase (ChAT). NENs in the human forebrain express nestin (green) and ChAT (red) in the basal nucleus of Meynert (**A**), nucleus of the diagonal band (**B**), and putamen (**C**). Vertical images taken through the YZ plane of the tissue section confirm the co-localization of nestin and ChAT evident in the XY plane of a given NEN. Scale bar: 30 µm.

We investigated some of these Class III-like cells further to determine if they expressed a protein associated exclusively with neurons. Aware that many of the neurons in the regions in which Class III-like cells are located in the human brain are cholinergic, we stained cells in the basal nucleus of Meynert, NDB, and putamen for nestin and ChAT. We determined that all Class III-like cells in each of these regions expressed both nestin and ChAT (Figure 12), strongly suggesting that these cells in the human brain are NENs, as in the rat brain. Although we did not stain nestin^+^ Class III-like cells in the supraoptic nucleus, paraventricular nucleus of the hypothalamus, septal nucleus, lateral division of the bed nucleus of the stria terminalis, or globus pallidus for neuronal markers, the marked structural similarity of these cells to neurons provides reasonable preliminary evidence to conclude cautiously that these Class III-like cells also are neurons that express nestin.

The density of NENs was particularly striking in the basal nucleus of Meynert, supraoptic nucleus, paraventricular nucleus of the hypothalamus, and NDB (Figures 11A–D). NENs in these regions frequently displayed strong nestin immunostaining in their cell somas and primary processes. By contrast, NENs appeared scattered in the septal nucleus, the lateral division of the bed nucleus of the stria terminalis, the globus pallidus and the putamen (Figures 11E–H). The cell somas of septal NENs (Figure 11E) were lightly stained as were their primary processes. NENs in the lateral division of the bed nucleus of the stria terminalis (Figure 11F) had large ovoid cell somas and sometimes displayed strong nestin staining in up to five primary processes. In the globus pallidus (Figure 11G) and putamen (Figure 11H) the density of NENs was low, as was also observed in the corpus striatum of the rat. Most of the NENs in the globus pallidus had cell somas that were approximately 20–25 µm in diameter and usually displayed one and sometimes two nestin stained processes. NENs in the putamen had cell somas with diameters that typically ranged from 25–35 µm. Nestin staining in primary processes was evident, but the staining usually was weak and rarely extended in any process more than 5 µm from the cell soma.

## Discussion

The expression of nestin by neural cells in the adult brain is significantly more widespread than has been previously thought. In this report we have described four distinct classes of nestin expressing neural cells in the adult rat brain, and have demonstrated that at least one of these classes of cells, Class III cells, exists in the human brain also. Class I cells, which are among the smallest neural cells in the rat brain, are distributed at low density throughout the forebrain. Class I cells proliferate locally in response to a cortical injury, suggesting that they can be triggered to divide by signals arising from injured cells. It is not unreasonable to speculate that Class I cells may be one of the sources of neural progenitor cells that can be derived in culture from the corpus striatum, the neocortex and other non-neurogenic regions of the adult brain [25], [26].

Class IV cells also are widely distributed throughout the forebrain. They are distinguished from any of the other classes of nestin expressing cells by their unique location, immediately adjacent to a neuron nearly 80% of the time. This proximate location suggests a possible supporting role for Class IV cells as has been proposed for satellite oligodendroglial cells, but immunocytochemical staining indicates that Class IV cells are not oligodendrocytes or their precursors. In contrast with the widespread distribution of Class I and Class IV cells, Class II cells appear to be located principally in the walls of the third ventricle, scattered in small clusters along the rostrocaudal length of the ventricle, the aqueduct, and in the medial wall of the lateral ventricle close to its merger with the third ventricle. Under normal conditions, Class II cells do not appear to divide, or do so at a rate that is so infrequent that a daily injection of BrdU (150 mg/kg) for seven days fails to label a single Class II cell. It remains to be determined whether Class II or Class IV cells are mitotically active in an injured brain.

Perhaps the most interesting and challenging nestin expressing cells in the adult forebrain are Class III cells, or, as we have termed them, nestin expressing neurons, NENs. Distributed principally in the cholinergic basal forebrain (rat and human) and closely associated regions such as the corpus striatum (rat) or basal ganglia (human) and hippocampus (rat), NENs, which coexpress both nestin and a range of proteins associated exclusively with neurons, present an apparent paradox in the normal adult brain. In contrast, in the abnormal human brain nestin-expression in neurons has been reported in various pathological states such as in the dentate gyrus of pediatric patients with temporal lobe epilepsy [27], in adult patients with cortical dysplasia and partial seizures [28], and in Purkinje cells of the cerebellum and in neurons in the frontal and temporal lobes of patients with Creutzfeldt-Jakob disease [29]–[31].

In the normal human brain, one report of nestin expression by apparent neurons has appeared [13]. However, the results in this report are based upon the expression of neuron-specific enolase (NSE) to identify a cell as a neuron, and NSE is not expressed exclusively by neurons. NSE expression has been reported in normal [32] as well as reactive and neoplastic astrocytes [33], and in oligodendrocytes [34]. Furthermore, Gu et al. [13] also reported that the corpus striatum and globus pallidus had very little nestin immunoreactivity, while we observed substantial numbers of NENs in the putamen and globus pallidus.

Using a more reliable marker, NeuN, to identify a cell as a neuron, Wang et al. [14] and Guo et al. [15] described the expression of nestin by neurons in the rostral basal forebrain of the adult rat, in particular the MSN and NDB, confirming some of the results presented here and reported previously. However, we have identified cells that are NENs in many other nuclei of the rat basal cholinergic forebrain, as mentioned, as well as in several related regions, such as the piriform cortex, corpus striatum and hippocampus.

A decline in the number of NENs in the MSN and NDB in juvenile (1 month old) and young adult (3 months old) rats compared with the number of NENs seen in very old rats (24 months old) has been reported [35]. Here we examined NENs in four human brains that ranged in age from 32–90 years at the time of death, but did not observe any obvious differences among the brains in the number of NENs that could be related to age. It is possible that this failure to detect an age-related decline in the number of NENs reflects a species difference between rats and humans. It also is possible that an age-related difference was not seen, because we studied only one brain at each age, and the variables that can affect the immunohistochemical staining of human brain tissue are significant. The quality of immunostaining of sections from the human brain is influenced by the interval that has elapsed between death and the fixation of the brain, the method used for fixation, and the quality of the resulting fixation. Unfortunately, in contrast to experimental studies, it is often difficult to control tightly for any of these variables when studying the human brain. Therefore, examining NENs in several brains at each of different ages will be necessary to determine whether the number of NENs in the basal forebrain of humans declines with age. Until this is done, our finding of no apparent decline in NENs with age in the human brain must be treated cautiously.

The results presented here raise the question of why a subset of neurons in the normal adult rodent and human basal forebrain and related regions express nestin, while the majority of neurons in the brain do not, and what is the role played by the nestin expression in these cells? In view of the selective distribution of NENs in the cholinergic basal forebrain and related regions of the brain that all are involved in higher order cognitive functions such as attention, learning and memory, it is reasonable to speculate that cell cycle and/or plasticity-related events may be implicated in the expression of nestin by NENs.

It is possible that NENs may be de-differentiating and expressing nestin prior to re-entering the cell cycle in preparation for dying or dividing. For two reasons this seems an unlikely possibility. First there is no evidence for increased cell death, as indicated by pyknotic cells or cells with condensed chromatin, in regions of the brain where NENs are observed than can be found in regions of the brain where NENs do not exist. Secondly, in detemining whether NENs are newly born, and, if, therefore, the expression of nestin reflects a phenotypic transition between a neural progenitor cell and terminally differentiated neuron, we did not observe a single BrdU-labeled NEN in rats that had received a daily injection of BrdU for 28 days prior to sacrifice, indicating that NENs in the normal rat brain had not re-entered the cell cycle and divided, nor had they arisen from the division of another cell.

However, the apparent lack of mitosis by NENs in the normal brain may not prevail in the injured brain. Of particular interest is the report of Nakatomi et al. [36] demonstrating the endogenous repopulation of degenerating pyramidal cells in the CA fields of the hippocampus following an ischemic insult. While the majority of new pyramidal cells appear to have migrated into the hippocampus from the posterior periventricle, some of the new pyramidal cells were generated locally, and may have arisen from the nestin-postive pyramidal cells that we have observed in the CA fields of the hippocampus.

Perhaps the expression of nestin by NENs is neuroprotective, because nestin may play a role in the stabilization of microtubules, which is often disrupted when neurons are injured or otherwise insulted. While our results with 192-Saporin do not support a neuroprotective role for the expression of nestin by NENs, these results are not adequate by themselves to rule out the possibility that nestin expression in some of the cholinergic neurons in the basal forebrain and related regions is neuroprotective. 192-Saporin is a powerful, rapidly acting toxin that targets cholinergic neurons and kills them by inactivating ribosomes. Perhaps, therefore, it is not surprising that nestin, an intermediate filament, is ineffective in blocking a cytotoxin that does not affect a cell's cytoskeleton. Future studies involving the targeting of cholinergic cells with a cytoskeletal disrupter will need to be conducted to explore whether nestin expression by cholinergic neurons in the basal forebrain is neuroprotective.

Alternatively, it is well known that in mitotically active cells, nestin plays an important role, regulated by phosphorylation, in the assembly and disassembly of intermediate filaments, which contributes to the remodeling of the cell's cytoskeleton. Nestin preferentially forms heterodimers with vimentin and alpha-internexin, possibly because these composites are more stable than nestin homodimers. This is suggested by the fact that nestin contains a short N-terminal, a domain known to be essential for filament protein assembly [2], and inhibits filament formation *in vitro* when present at concentrations greater than 50% [37]. Thus, nestin may aid in linking intermediate filaments with each other, and with microtubules and microfilaments via nestin's long C-terminal domain. Throughout the cell cycle, nestin colocalizes with the intracellular reorganization of vimentin filaments and aggregates, and is essential for the mitotic disassembly of vimentin [38]. This suggests a role for nestin-mediated reorganization of the cytoskeleton during mitosis that is mediated in part by the upregulation of phosphorylation of Thr^316^ by cdc2 kinase [39]. It is not unreasonable, therefore, to propose that at any given time a subset of cholinergic neurons in the basal forebrain and glutamatergic neurons in the hippocampus, NENs, are remodeling their cytoskeletons, because of the role these cells play in higher brain function, and that the expression of nestin by these cells reflects this remodeling.

Future studies will be needed to address why the cholinergic basal forebrain and closely related regions, and hippocampus, which undergo significant neuronal loss in Alzheimer's disease [40], support many NENs. The degeneration of aged neurons in the cholinergic basal forebrain in Alzheimer's disease (AD) is marked by synaptic dysfunction [41], [42], the deposition of amyloid plaques and the cytoskeletal disorganization of affected cells [43]–[49]. Young neurons are not at risk for AD, and it is possible that NENs also may be resistant to AD, because nestin's long non-α-helical carboxy terminal may play a role in the stabilization of microtubules [50]. Such stabilization is frequently disrupted in cholinergic neurons in AD by the abnormal phosphorylation of tau protein, resulting in a loss of function caused by the disruption of axonal transport. Determining whether the expression of nestin in basal forebrain cholinergic neurons in humans protects them from insults arising from microtubule disruption such as occur in AD would provide evidence for a neuroprotective role for nestin in otherwise mature cholinergic neurons. Understanding the role played by NENs in the cholinergic basal forebrain and related areas may help to shed light on the dynamics of neuronal degeneration in AD.

## Supporting Information

Figure S1 **Reduction of autofluorescence in cells in the human brain.** Neurons in the adult human brain contain lipofuscin, which fluoresces brightly over a wide range of illuminating wavelengths and creates significant interference for immunohistochemical studies. Autofluorescence was diminished significantly after treatment with a Sudan Black solution (Millipore). Autofluorescing cells in an untreated section of the human brain (**A**), a brain section treated with Sudan Black (**B**), and cells stained for ChAT and visualized with a Cy3-conjugated secondary antibody in a section treated with Sudan Black (**C**). All images were collected under identical confocal microscopy settings. Scale bar: 100 µm.
(TIF)

Figure S2 **Nestin expressing cells in the subventricular zone (SVZ) and rostral migratory stream (RMS).** Immunostaining of coronal sections from the adult rat brain showing dense nestin expression in the SVZ (**A**) and in the RMS as it courses over the dorsal surface of the corpus striatum (**B**). Cells leaving the SVZ and entering the RMS stained for nestin (green) and BrdU (red). Note that many of the cells express nestin and are labeled with BrdU (yellow). Scale bar: 200 µm (**A**), 100 µm (**B**), 50 µm (**C**).
(TIF)

Figure S3 **Selective immunolesioning of p75(NGFR) nerve growth factor receptor expressing cholinergic neurons with 192-Saporin.** p75(NGFR) staining in the nucleus of the diagonal band (NDB) of a control rat (**A**) and in the NDB of a rat that received an intraventricular injection of 2 µg of 192-Saporin in 6 µL of PBS and survived for six days (**B**). Diagram (**C**) showing the locations of the fields that were selected for imaging and quantifiying the cytotoxic effects of 192-Saporin administration on p75(NGFR) expressing cholinergic neurons in the medial septal nucleus (**MSN**) and the nucleus of the diagonal band (**NDB**) (See text Figure 10, panel D). Scale bar: 100 µm.
(TIF)

Figure S4 **Distribution of NENs in the human forebrain.** Coronal drawings of the human basal forebrain from Mai et al. [51]. NENs (black dots) were found throughout the basal forebrain, including the basal nucleus of Meynert (**BC**), external globus pallidus (**EGP**), external lamina of the globus pallidus (**lml**), interomedial septal nucleus (**LSI**), lateral caudate nucleus (**CdL**), lateral division of the bed nucleus of the stria terminalis (**BSTL**), medial caudate nucleus (**CdM**), nucleus of the diagonal band **(DB**), paraventricular hypothalamic nucleus (**Pa**), putamen (**Pu**), supraoptic nucleus (**SO**) and ventrolateral septal nucleus (**LSV**).
(TIF)
