# Supplementary figures and images for: Expression of Nestin by Neural Cells in the Adult Rat and Human Brain
Source: PLoS One. 2011 Apr 7;6(4):e18535. doi: 10.1371/journal.pone.0018535 (PMC3072400; doi:10.1371/journal.pone.0018535)

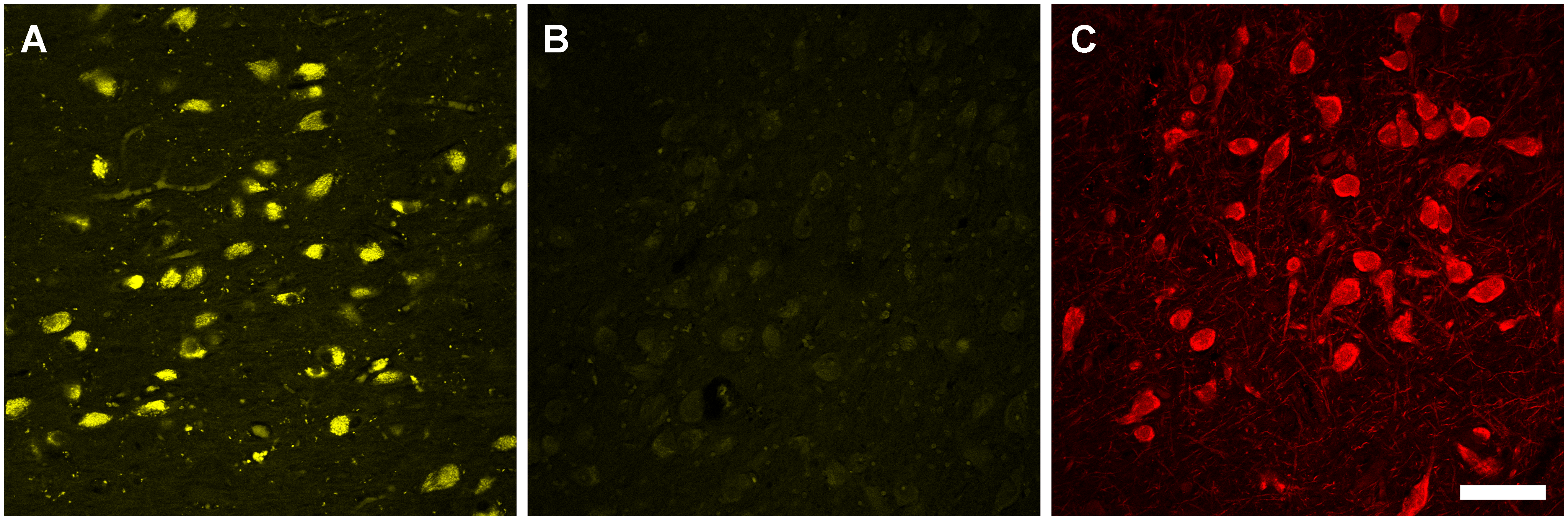

Supplement: Figure S1 — Reduction of autofluorescence in cells in the human brain. Neurons in the adult human brain contain lipofuscin, which fluoresces brightly over a wide range of illuminating wavelengths and creates significant interference for immunohistochemical studies. Autofluorescence was diminished significantly after treatment with a Sudan Black solution (Millipore). Autofluorescing cells in an untreated section of the human brain (A), a brain section treated with Sudan Black (B), and cells stained for ChAT and visualized with a Cy3-conjugated secondary antibody in a section treated with Sudan Black (C). All images were collected under identical confocal microscopy settings. Scale bar: 100 µm. (TIF) [file pone.0018535.s001.tif]

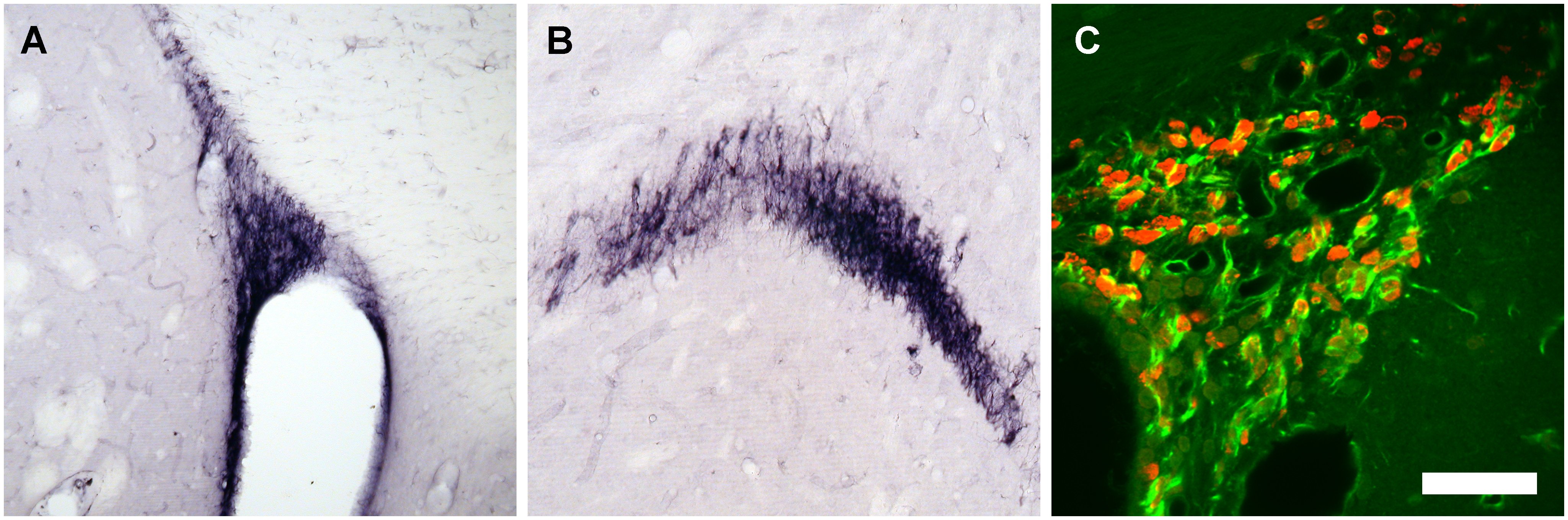

Supplement: Figure S2 — Nestin expressing cells in the subventricular zone (SVZ) and rostral migratory stream (RMS). Immunostaining of coronal sections from the adult rat brain showing dense nestin expression in the SVZ (A) and in the RMS as it courses over the dorsal surface of the corpus striatum (B). Cells leaving the SVZ and entering the RMS stained for nestin (green) and BrdU (red). Note that many of the cells express nestin and are labeled with BrdU (yellow). Scale bar: 200 µm (A), 100 µm (B), 50 µm (C). (TIF) [file pone.0018535.s002.tif]

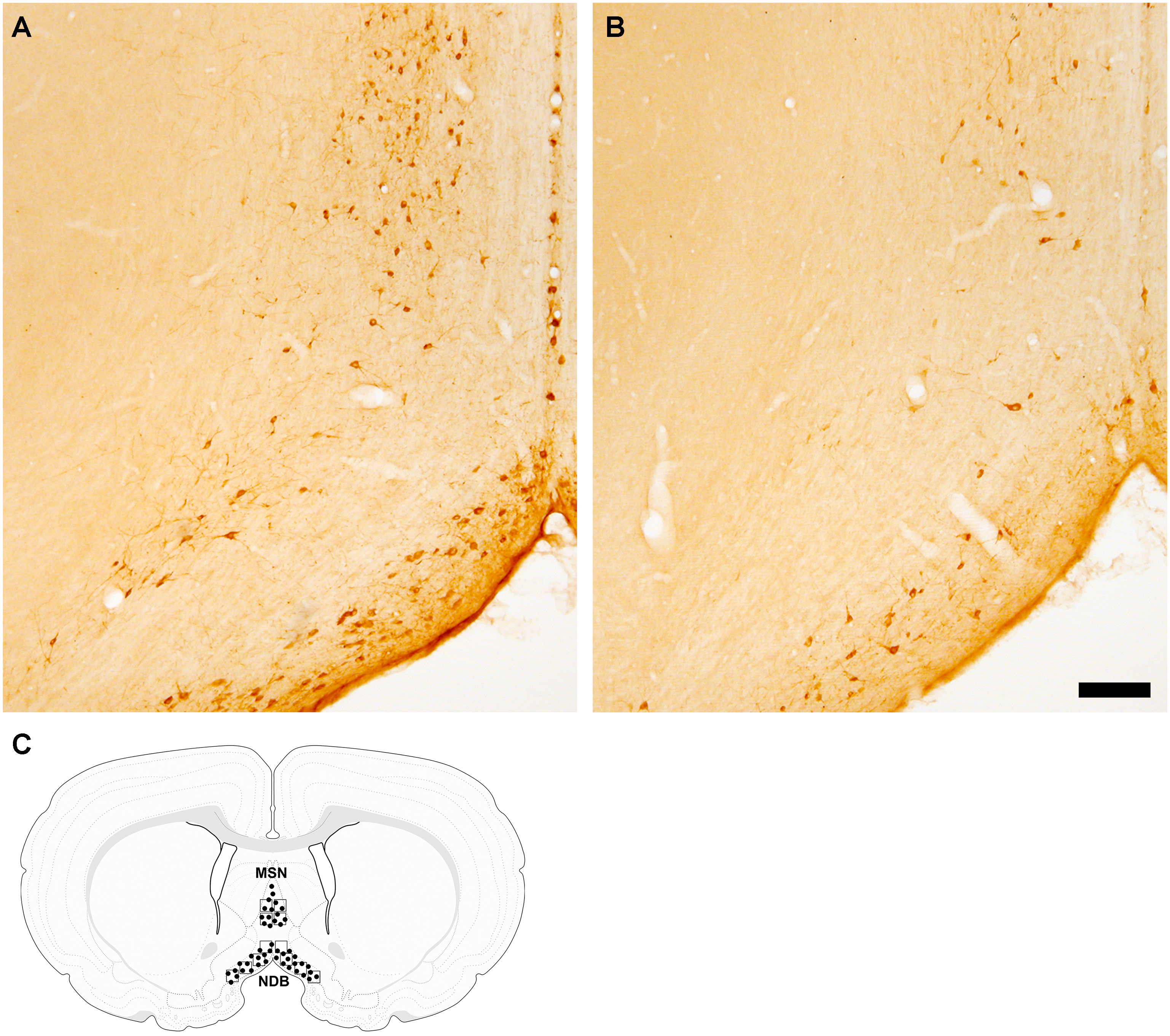

Supplement: Figure S3 — Selective immunolesioning of p75(NGFR) nerve growth factor receptor expressing cholinergic neurons with 192-Saporin. p75(NGFR) staining in the nucleus of the diagonal band (NDB) of a control rat (A) and in the NDB of a rat that received an intraventricular injection of 2 µg of 192-Saporin in 6 µL of PBS and survived for six days (B). Diagram (C) showing the locations of the fields that were selected for imaging and quantifiying the cytotoxic effects of 192-Saporin administration on p75(NGFR) expressing cholinergic neurons in the medial septal nucleus (MSN) and the nucleus of the diagonal band (NDB) (See text Figure 10, panel D). Scale bar: 100 µm. (TIF) [file pone.0018535.s003.tif]

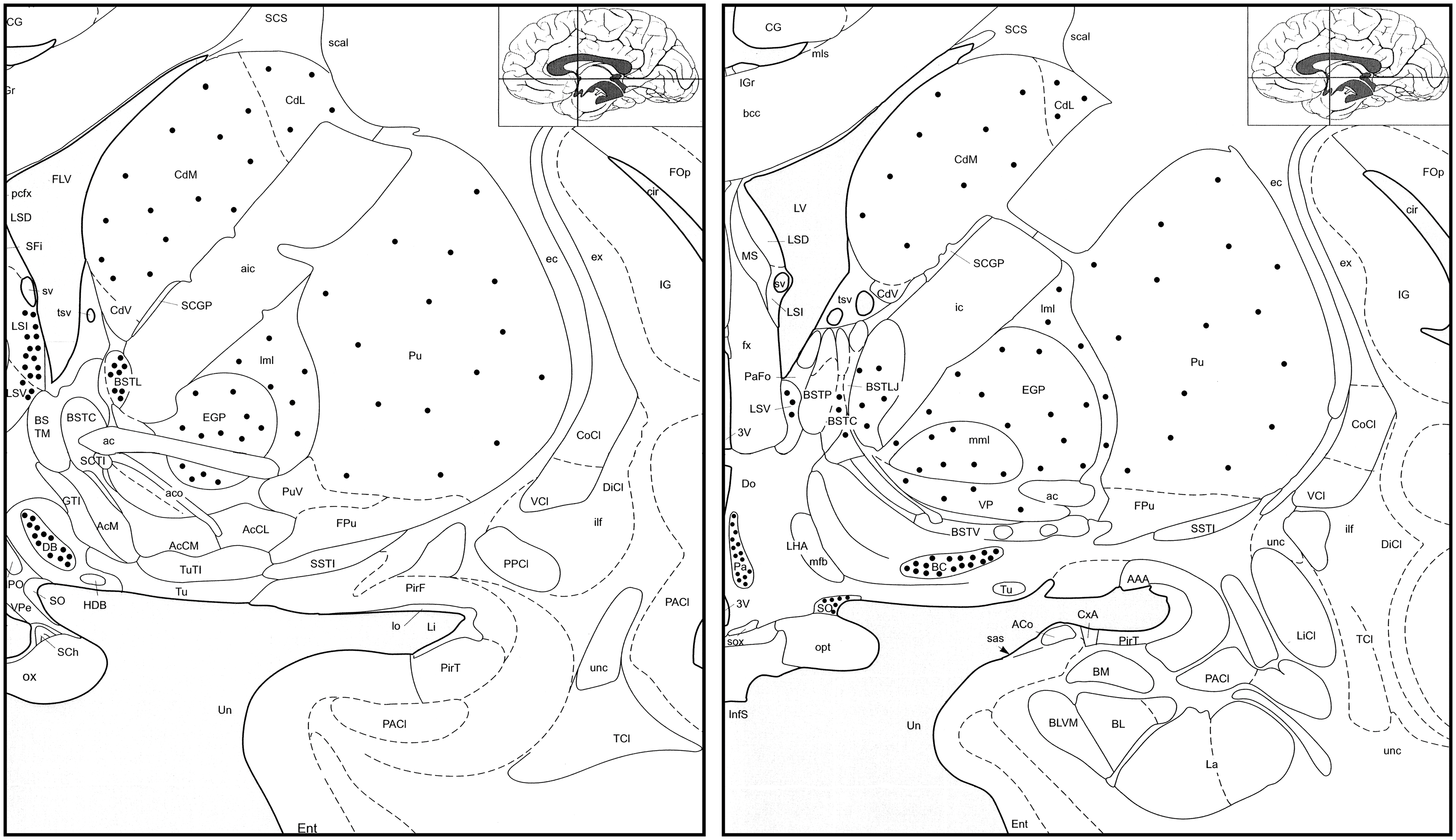

Supplement: Figure S4 — Distribution of NENs in the human forebrain. Coronal drawings of the human basal forebrain from Mai et al. [51]. NENs (black dots) were found throughout the basal forebrain, including the basal nucleus of Meynert (BC), external globus pallidus (EGP), external lamina of the globus pallidus (lml), interomedial septal nucleus (LSI), lateral caudate nucleus (CdL), lateral division of the bed nucleus of the stria terminalis (BSTL), medial caudate nucleus (CdM), nucleus of the diagonal band (DB), paraventricular hypothalamic nucleus (Pa), putamen (Pu), supraoptic nucleus (SO) and ventrolateral septal nucleus (LSV). (TIF) [file pone.0018535.s004.tif]
